# Male sexual dysfunction in Ireland: prevalence and associated sociodemographic characteristics

**DOI:** 10.1186/1753-6561-6-S4-O27

**Published:** 2012-07-09

**Authors:** DR Walsh

**Affiliations:** 1 Department of Psychology, Division of Population Health Sciences, Royal College of Surgeons in Ireland

## Introduction

Sexual problems and dysfunctions are relatively common experiences which significantly impact on the perceived quality of life; sexual dysfunction has been shown to be associated with anxiety, depression, interpersonal difficulties and overall negative well-being. Recent research has shown that sexual health can be indicative of overall health, for example; erectile dysfunction has been shown to be a sentinel marker of cardiovascular disease. The aims of this report were to assess the prevalence of male sexual dysfunction in Ireland and to identify sociodemographic factors associated with sexual dysfunction.

## Methods

Data collected in 2003 from the Irish Study of Sexual Health and Relationships (ISSHR) was analyzed using PASW statistics 18. In the survey, men aged 18-64 were asked questions about sexual problems which lasted “for at least one month in the past 5 years.” A score variable (MSD) was computed for the number of dysfunctions each participant had reported ranging from zero to six. PASW was used to examine any associations between the continuous MSD variable and fertility, marital status, STI history or age of first sex.

## Results

At least one sexual dysfunction was prevalent in 49.6% of men who participated in the survey. Erectile dysfunction was reported by 15.4% of men and premature ejaculation by 24.4% of men. Dyspareunia and anorgasmia both had a higher than expected prevalence of 16.1% and 14.9% respectively. The most common sexual problem was low sexual desire with a prevalence of 30.2%. Marital status and fertility were found to have no significant association with MSD. Having previously had an STI and having had first sexual intercourse at a young age (especially 16 years or younger) were both correlated with an increased prevalence of MSD.

## Conclusions

Male sexual dysfunction has a high prevalence in the Irish population. Predictors of MSD such as past STI infections could help healthcare providers detect the patients most likely to experience MSD. More research is required on the epidemiology of and risk factors associated with anorgasmia and dyspareunia. Understanding the prevalence of and factors associated with sexual problems will improve the provision of care and support available.

